# Understanding Vaccine Hesitancy in the Context of COVID-19: The Role of Trust and Confidence in a Seventeen-Country Survey

**DOI:** 10.3389/ijph.2021.636255

**Published:** 2021-05-14

**Authors:** Laura S. Rozek, Pauline Jones, Anil Menon, Allen Hicken, Samantha Apsley, Elizabeth J. King

**Affiliations:** ^1^ Department of Environmental Health Sciences, School of Public Health, University of Michigan, Ann Arbor, MI, United States; ^2^ Department of Political Science, College of Literature, Sciences and the Arts University of Michigan, Ann Arbor, MI, United States; ^3^ Department of Health Behavior and Health Education, School of Public Health, University of Michigan, Ann Arbor, MI, United States

**Keywords:** COVID-19, vaccine hesistancy, trust, global health, public health

## Abstract

**Objectives:** An effective vaccine to SARS-CoV-2 cannot be successfully deployed if a significant number of people worldwide are unwilling to accept it. We investigated the relationship between trust in scientists and medical professionals and perceptions of vaccine safety and effectiveness. We also build on past studies by exploring the relationship between confidence in global health organizations and vaccine hesitancy.

**Methods:** We conducted an online survey in seventeen countries/territories across five world regions between May -June 2020. We assessed the relationship between COVID19 vaccine hesitancy, confidence in public health organizations, and trust in key experts and leaders.

**Results:** Our findings strongly suggest that confidence in the World Health Organization combined with trust in domestic scientists and healthcare professionals is a strong driver of vaccine acceptance across multiple countries/territories.

**Conclusion:** We find that hesitancy is widespread, and uptake would be insufficient to achieve herd immunity. There is widespread confidence in how public health organizations have responded to the current pandemic and this is related to vaccine acceptance. Our results also highlight the important role of trust in health care providers and scientists in reducing COVID19 vaccine hesitancy.

## Introduction

With the realization that a vaccine to SARS-CoV-2 is essential to gain control of the pandemic, many have expressed legitimate concerns about vaccine hesitancy [1, 2]. Vaccine hesitancy, defined as the delayed acceptance of or refusal to accept an available vaccine [3], is a global problem that is on the rise. Just a year before the SARS-CoV-2 pandemic, vaccine hesitancy was considered one of the top 10 global health threats [4]. Country-specific longitudinal trends prior to the COVID-19 pandemic show the impact of anti-vaccine movements and misinformation on vaccine hesitancy in some countries and regions [4]. These results indicate that much of the world falls short of effective vaccine acceptance levels for COVID-19 herd immunity, especially when there are concerns about vaccine safety [5, 6]. The reluctance to receive a vaccine during a pandemic is not without precedent, as illustrated by the large portion of adults who refused to be vaccinated during the 2009 H1N1 influenza pandemic [7]. An effective vaccine for COVID-19 cannot be successfully deployed if a significant number of people worldwide are unwilling to accept it. Given the global nature of COVID-19, it is essential to understand the dynamics of vaccine hesitancy in different countries and contexts, to fully appreciate the reasons for hesitancy, and to develop strategies and interventions for achieving herd immunity [8].

Although most scholars agree that the primary drivers of vaccine hesitancy are often context-specific, there is some consensus that confidence and trust play a critical role in reducing vaccine hesitancy across contexts [9, 10], particularly when it comes to public healthcare providers [11]. Concerns about vaccine hesitancy during the current SARS-CoV-2 pandemic, for example, were partially attributed to the decline in trust in science and medicine across the globe [12]. Conversely, recent research, prior to the COVID-19 pandemic, has found not only that trust in scientists and healthcare professionals is higher than expected around the world, but also that there is a strong relationship between trust in scientists and medical professionals and perceptions of vaccine safety and effectiveness [13]. While this is good news, there remain important gaps in our understanding of what types of trust and confidence are most critical to vaccine acceptance, how this varies across countries, and the role of trust and confidence in the context of a global pandemic.

Our multidisciplinary group conducted a seventeen-country survey to address gaps in our knowledge of vaccine hesitancy by investigating the relationship between vaccine hesitancy and different types of trust and confidence. Whereas previous studies have focused on trust and confidence in scientists and domestic healthcare professionals, we explore whether trust and confidence in other key domestic actors, including politicians and religious leaders affect vaccine hesitancy. We also build on past studies by exploring the relationship between confidence in global, national, and local health organizations and vaccine hesitancy. Our cross-national survey provides some insight into how the relationship between different kinds of trust and confidence and vaccine hesitancy varies across countries. These findings could help inform context-specific approaches to addressing vaccine hesitancy. Because our survey was conducted during the first wave of the SARS-CoV-2 pandemic (May-June 2020), moreover, it adds critical insight into our understanding of how trust and confidence is linked to vaccine hesitancy early on in a pandemic.

## Methods

### Study Design and Sampling: COVID19 Studying International Coping and Compliance Survey

We conducted an online cross-sectional survey in seventeen countries/territories across five world regions: North America (Canada, United States); Europe/Eurasia (Germany, Poland, Russia, Sweden, Ukraine); East Asia (China, Hong Kong, Taiwan); Southeast Asia (Indonesia, Malaysia, Philippines, Singapore, Thailand, Vietnam); and the Middle East (Turkey). In order to maximize the diversity of our sample, we included countries and territories in our survey that varied across several important factors–their level of economic development, dominant culture, regime type, and government response to the pandemic–while also taking into consideration the area expertize of our research team. The survey was translated into the respective local languages for each country. For all seventeen countries/territories included in the analysis, we used the Qualtrics online survey platform due to the constraints against conducting in-person surveys in the context of a pandemic. For sixteen of our countries, Qualtrics maintains a country-level database of residents who have volunteered to participate in survey-based research from which Qualtrics recruits survey respondents via Qualtrics panels, enabling us to achieve high response rates (www.qualtrics.com). Panel research is a rapid method for collecting data repeatedly, drawing a sample from a pre-recruited set of respondents. Qualtrics maintains a list of pre-qualified and willing group of respondents to participate in surveys on an as-needed basis worldwide. For sixteen of these countries/territories, we used quota sampling methods to target a Qualtrics panel sample that was representative of the country’s demographics with respect to age and gender. In Russia, participants were recruited exclusively using web-based recruitment via snowball sampling due to Qualtrics’ inexperience recruiting participants in that country. This approach is in accordance with the policies of the University of Michigan’s Institutional Review Board. Of the 36,546 people who accessed the Qualtrics landing page and reviewed the consent form, 17,158 (41.3%) completed the survey via Qualtrics samples and 450 completed the survey through snowball sampling (Russia). Data were collected between May 21 and June 24, 2020.

### Data Analysis

For the purpose of these analyses, we measured three key variables: vaccine hesitancy; confidence in public health organizations; and trust in several professional and leadership figures. Table 1 provides details regarding the verbatim questions and response options used to measure each of these variables.^1^

**TABLE 1:** Main independent and dependent variables used in analyses, COVID19 Studying International Coping and Compliance Survey, 2020.

Measure	Survey prompt	Levels
Confidence in public health organizations	“In general, how much confidence do you have in the following organizations’ information about and handling of the coronavirus?”	1. none at all
	1) international (“the world health organization” (WHO))	2. not very much
	2) national (“my country’s national health organization or ministry of health”)	3. some confidence
	3) local (“my local health department”)	4. a lot of confidence
Trust in key experts and leaders	“In general, how much do you trust the following groups of people?”	1. do not trust at all
	1) medical practitioners	2. do not trust very much
	2) scientists	3. trust somewhat
	3) political leaders	4. trust completely
	4) religious leaders	
Socioeconomic status	“Which of the following statements most accurately reflects the financial situation of your family before COVID-related policies took effect?”	1. We do not have enough money for food
		2. We have enough money for food, but not enough money for clothes
		3. We have enough money to buy food and clothes, but not enough to buy expensive items, such as a TV or refrigerator
		4. We have expensive items, such as a new TV or refrigerator, but no car
		5. We can buy almost anything we want

We estimate the relationship between the confidence and trust measures and vaccine hesitancy using ordinal logistic regressions. These multivariable models also include age, education, socioeconomic status, gender, and country-fixed effects. Age and gender were self-reported by the respondent. Socioeconomic status was determined by self-reported measures of financial well-being (Table 1). We conducted all our analyses using R statistical software (The R Foundation, 3.6.3). The total number of observations utilized in the regression (15,151) is slightly lower than the number of respondents for each question because any respondent for whom a value is missing across any of the variables included in the analysis is dropped when estimating marginal effects.

### Role of Funding Source

The funding source has no involvement is the design, collection, analysis, or interpretation of these data.

## Results

### COVID-19 Vaccine Hesitancy

**FIGURE 1:**
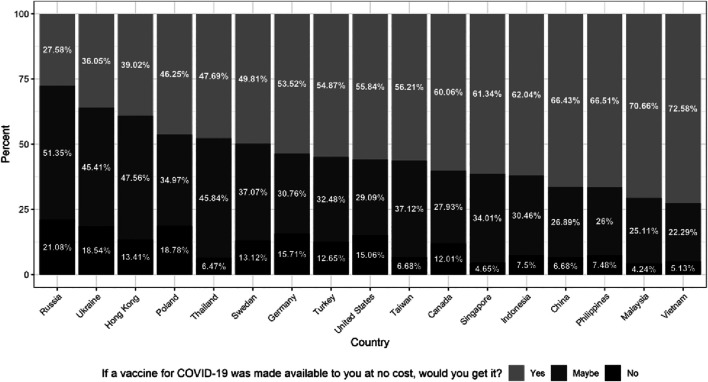
Vaccine hesitancy by country (left to right–highest to lowest). Note: This figure summarizes individual responses (yes, maybe, no), by country, to the following question: “If a vaccine for COVID-19 was made available to you at no cost, would you get it?” The countries are arranged from highest to lowest percentage of respondents reporting either “maybe” or “no”. Sample size by country: Russia = 446; Ukraine = 1068; Hong Kong = 574; Poland = 1081; Thailand = 1082; Sweden = 1052; Germany = 1050; Turkey = 1099; United States = 1155; Taiwan = 749; Canada = 1074; Singapore = 538; Indonesia = 1080; China = 1138; Philippines = 1096; Malaysia = 944; Vietnam = 1014. COVID19 Studying International Coping and Compliance Survey, 2020.

Almost half the respondents (44%) responded either “no” or “maybe” when asked if they would take a COVID-19 vaccine (Figure 1). Women were more likely to be vaccine hesitant (46.46%) compared to men (42.26%). Older respondents (>70 years) were less likely to be vaccine hesitant (36.93%) compared to those less than 70 years of age (44.84%). Forty-one percent of respondents at the highest level of economic wellbeing reported vaccine hesitancy, compared to 48% of those who reported the lowest levels of economic wellbeing. As depicted in Figure 1, however, the degree of vaccine hesitancy varies significantly by country/territory. The proportion of respondents answering “no” or “maybe” ranged from 72% to 27%.

We also find significant variation within the vaccine hesitant group by country/territory–specifically, in the number of respondents answering “maybe” vs. those answering “no” (Figure 1). The proportion that responded “maybe” is of particular importance because it suggests which countries/territories have a sizable fraction of the population that is undecided, and thus, can possibly be swayed to take the vaccine by investing in context-appropriate public health campaigns. The proportion of respondents answering “maybe” ranged from 51% to 22%. Thus, the pattern of “maybe” reflects the common pattern we found across countries/territories. Those with the highest proportion of “maybe” respondents also tended to have the highest proportion responding “no,” with the exception of three Asian countries/territories–Thailand, Taiwan, and Singapore.

### Confidence in Public Health Organizations

Across the countries/territories in our sample, confidence in public health organizations with respect to information about and handling of the coronavirus is relatively high (see Figure 2a), though it varies by the level at which the organization operates. At the international level, the majority of countries/territories have a high proportion of respondents (greater than 70%) that express either “some confidence” or “a lot of confidence” in the WHO. In only two countries/territories does the proportion of respondents expressing confidence in the WHO fall below 50%: Hong Kong (48%) and Taiwan (22%). At the national level, the proportion of respondents in the majority of countries/territories that express either “some confidence” or “a lot of confidence” in public health organizations is even higher (greater than 75%). In only one country (Russia) does the proportion of respondents expressing confidence in the national health ministry fall below 50%, and in only four other countries/territories do they fall below 75% (Hong Kong, Philippines, Poland, and Ukraine). Finally, at the local level, the proportion of respondents that express either “some confidence” or “a lot of confidence” in the local health department ranges from 29% (Russia) to 91% (Vietnam). In most countries/territories a high proportion of respondents (greater than 70%) express either “some confidence” or “a lot of confidence” in the local health department.

**FIGURE 2A:**
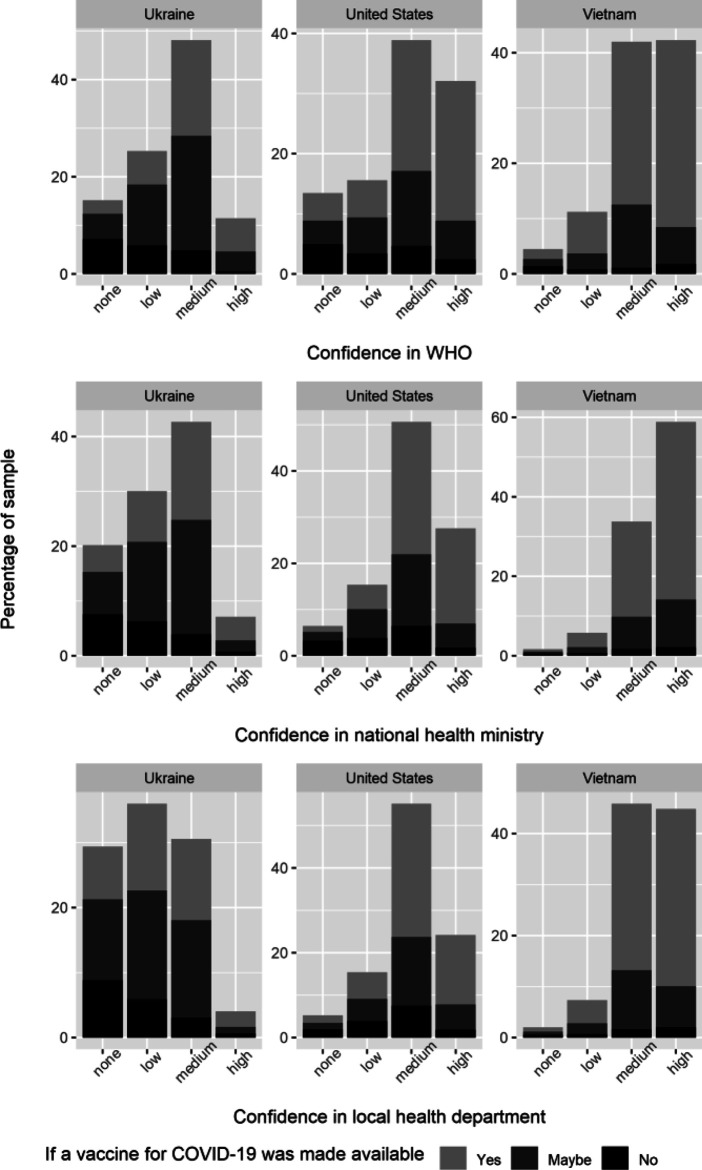
Measures of confidence, representative countries. Note: This figure depicts the distribution of confidence (none at all, not very much, some confidence, a lot of confidence) across respondents, by country, in the World Health Organization, National Health Ministry, and Local Health department, respectively. This figure also provides information on vaccine hesitancy across differing levels of trust and confidence. To measure vaccine hesitancy, we asked individuals to respond (yes, maybe, or no) to the following question: “If a vaccine for COVID-19 was made available to you at no cost, would you get it?” We picked the three countries above to illustrate the distribution of confidence measures in countries with high (Ukraine, 64%), medium (United States, 45%), and low (Vietnam, 27%) vaccine hesitancy, respectively. COVID19 Studying International Coping and Compliance Survey, 2020.

We also found that in most countries/territories, confidence in the national health ministry and local health department is higher than it is in the WHO. There are only four exceptions (WHO vs. national ministry of health and local health department): the Philippines (75% vs. 66% and 63%); Poland (68% vs. 56% and 51%); Russia (63% vs. 40% and 29%); and Ukraine (59% vs. 50% and 35%). For most countries/territories, moreover, the gap between expressed confidence in the WHO and expressed confidence in the national ministry of health and local health department is relatively small (less than 10 percentage points), with the smallest gaps in China and Indonesia. Only in two countries/territories–Singapore and Taiwan–is this gap greater than 10%.

### Trust in Experts and Leaders

There is much greater variation when it comes to trust in experts and leaders across the countries/territories in our sample (see Figure 2b). In most countries/territories, the majority of respondents expressed trust in medical professionals, such as doctors and nurses, as well as scientists. The proportion of respondents answering that they trust medical professionals either “somewhat” or “completely” was almost universally greater than 80%. In several countries/territories, including the United States, this proportion was greater than 90%. Ukraine is the only country in which the proportion of respondents that expressed trust in medical professionals fell below 80%, and yet, it was still well over a majority (71%). Similarly, the proportion of respondents answering that they trust scientists either “somewhat” or “completely” was greater than 80% across countries/territories in our sample with the exception of a few countries/territories (Malaysia, Taiwan, and Ukraine). In each of these countries/territories, however, this proportion was still well over a majority (73%, 78%, and 75%, respectively). In all countries/territories, except Ukraine, respondents have slightly less trust in scientists compared to medical professionals.

**FIGURE 2B:**
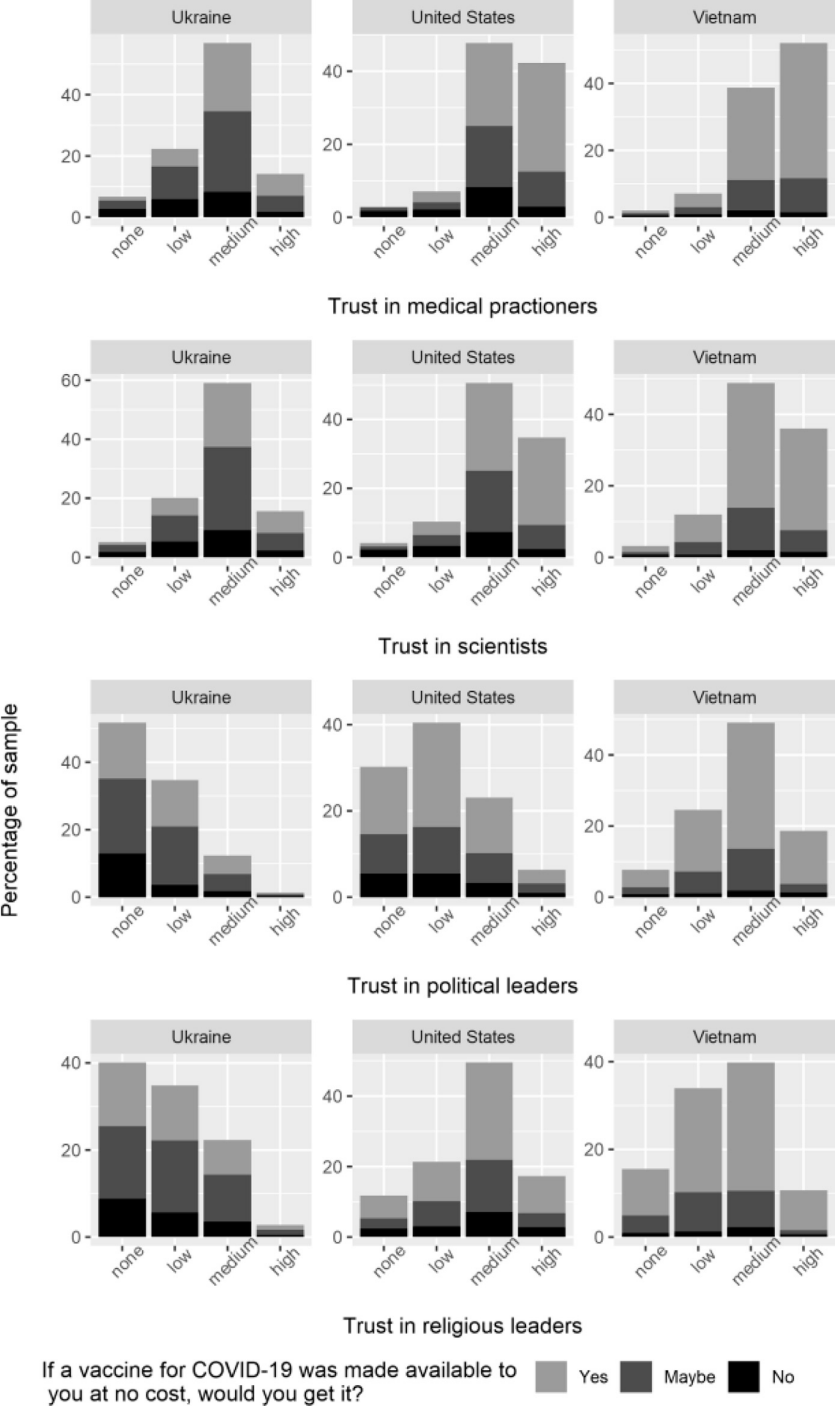
Measures of trust, representative countries. Note: This figure depicts the distribution of trust (do not trust at all, do not trust very much, trust somewhat, trust completely) across respondents, by country, in medical practitioners, in scientists, political leaders, and religious leaders, respectively. This figure also provides information on vaccine hesitancy across differing levels of trust and confidence. To measure vaccine hesitancy, we asked individuals to respond (yes, maybe, or no) to the following question: “If a vaccine for COVID-19 was made available to you at no cost, would you get it?” We picked the three countries above to illustrate the distribution of confidence measures in countries with high (Ukraine, 64%), medium (United States, 45%), and low (Vietnam, 27%) vaccine hesitancy, respectively. COVID19 Studying International Coping and Compliance Survey, 2020.

Conversely, in most countries/territories, a majority of respondents expressed a lack of trust in politicians and religious leaders, albeit much more so for the former than the latter. The proportion of respondents answering that they either “do not trust very much” or “do not trust at all” when asked about politicians was greater than 65% in two-thirds of the countries/territories included in our survey. In the remaining one-third, there are only two countries in which less than the majority do not have trust in politicians: China (44%) and Vietnam (32%). The proportion of respondents answering that they either “do not trust very much” or “do not trust at all” when asked about religious leaders was smaller in the majority of countries/territories and had a wider range. Although it falls above 50% in half the countries/territories in our sample, this proportion ranges from as low as 16% in Indonesia and 31% in Malaysia to as high as 79% in Sweden and 80% in Germany. In most countries/territories the proportion of respondents that expressed a lack of trust in politicians is greater than the proportion that expressed a lack of trust in religious leaders. The only exceptions are China, Germany, Sweden, and Vietnam.

### Relationship Between Confidence and Trust and Vaccine Hesitancy

**FIGURE 3:**
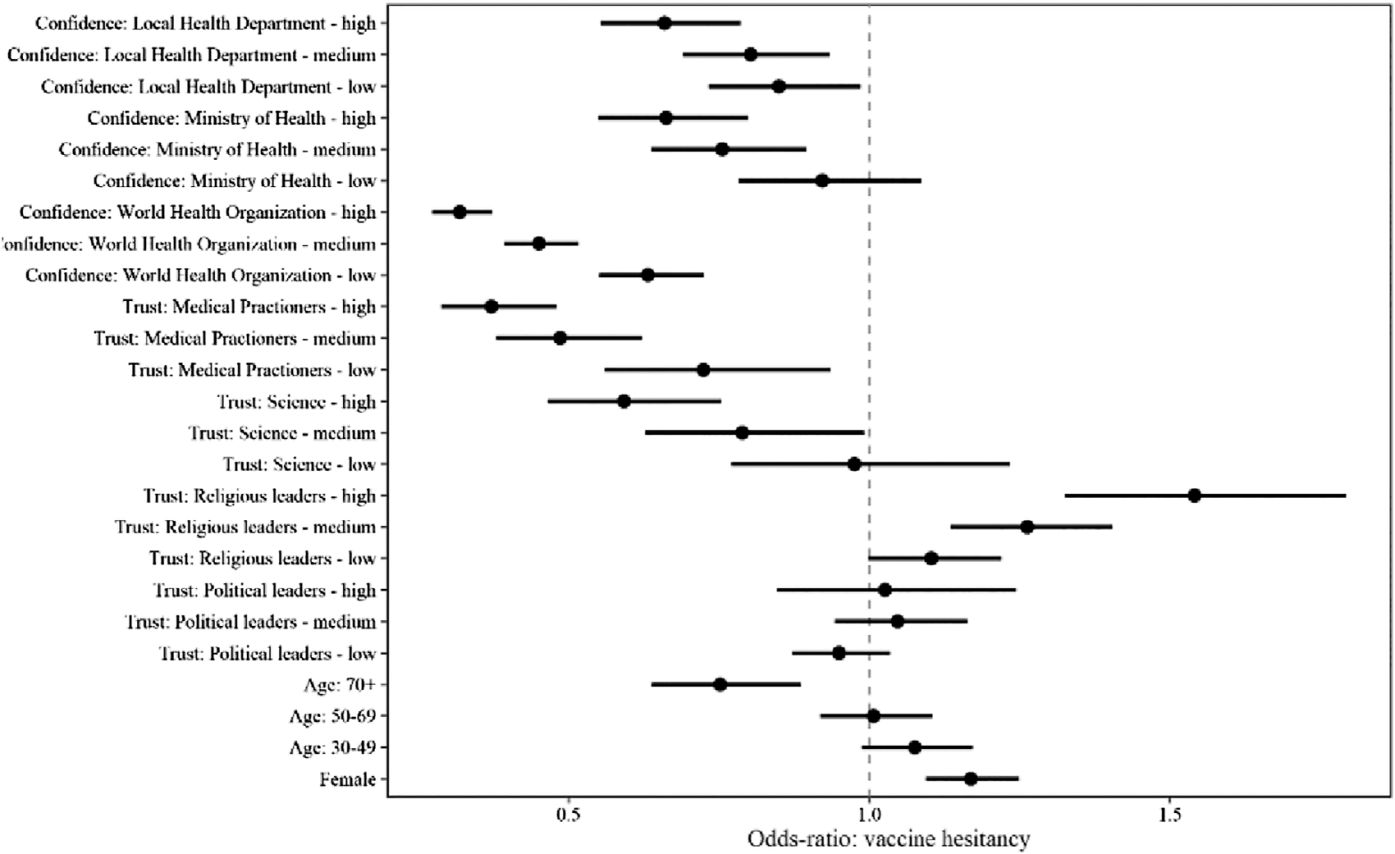
Multivariable modeling of confidence, trust, and vaccine hesitancy. Note: Results from estimating the relationship between confidence and trust measures and vaccine hesitancy using ordinal logistic regression. This multivariable model also includes age, education, socioeconomic status, gender, and country-fixed effects. Age and gender were self-reported by the respondent. Socioeconomic status was determined by self-reported measures of financial well-being. COVID19 Studying International Coping and Compliance Survey, 2020.

We report results from estimating the relationship between our confidence and trust measures and vaccine hesitancy (Figure 3). The strongest predictors of vaccine hesitancy were confidence in the WHO and trust in health practitioners; even those with low confidence in the WHO were 1.5 times less likely to express vaccine hesitancy compared to those who reported no confidence in the WHO. Meanwhile, those with a lot of confidence in the WHO were almost three times less likely to express vaccine hesitancy compared to those who reported no confidence in the WHO. Measures of trust in scientists, local health departments, and national health ministries were also predictive of reduced vaccine hesitancy. Higher trust in religious leaders was predictive of increased vaccine hesitancy [OR high/none = 1.54, *p* < 0.001, medium/none = 1.26, *p* < 0.001], while trust in politicians was not predictive. Meanwhile, respondents over the age of 70 were less likely to be vaccine hesitant when compared to respondents below 30 years of age [OR = 0.75, *p* < 0.001]. Women were more likely to be vaccine hesitant compared to men [OR = 1.17, *p* < 0.001]. Respondents indicating the highest level of economic wellbeing reported less vaccine hesitancy compared to those respondents indicating the most economic hardship [OR = 0.78, *p* < 0.001].

## Discussion

Our simultaneous survey of 17 countries/regions provides important insight into how trust and confidence play a role in vaccine hesitancy in various contexts. We know that there are specific historic factors that influence vaccine hesitancy. Obviously, the debunked Wakefield study is associated with widespread vaccine hesitancy and subsequent measles outbreaks worldwide, notably in the United States and France [14, 15]. Vaccine hesitancy can also be driven by regional factors. For example, the health risks associated with the dengue vaccine *Dengvaxia* in 2017 is largely believed to be the reason for increasing vaccine hesitancy in Indonesia and throughout the region [9]. However, previous studies have found trust and confidence to be an important factor across contexts [10, 16]. Our analysis contributes to these studies by examining what types of trust and confidence are most critical to vaccine acceptance, how this varies across countries, and the role of trust and confidence in the context of a global pandemic. Addressing these gaps in our knowledge is vital during the current pandemic, not only given the global rise in vaccine hesitancy [4, 16], but also given the fact that newer vaccines seem to generate greater hesitancy [17].

These data have several strengths. First, the timing of the survey–late May thru mid- June 2020–coincides with most of these countries/territories being in the midst or recently out of containment measures, including quarantine. Thus, this study provides early data that can be added to other surveys conducted later in the pandemic to provide a full complement of vaccine hesitancy data throughout the uncertain course of the pandemic. Second, our study uses a validated measure of vaccine hesitancy in seventeen countries/territories representing diverse regions of the world. Third, we examine trust and confidence in difference types of leaders and organizations, including politicians, religious leaders and the WHO, as well as domestic healthcare providers. At the same time, our findings are limited to a one-time assessment. It is likely that vaccine hesitancy is a dynamic process that is affected by country-level social and political conditions that may result in changes in hesitancy over time. The vital importance of vaccine hesitancy, in the context of COVID-19 and future potential pandemics, warrants assessment at several timepoints, with our data providing an essential early “snapshot” that can be built upon as the pandemic continues to progress.

Trust in government is a critical component to any public health measures and was at a historic low prior to the COVID-19 pandemic. Only 45% of citizens in Organization for Economic Co-operation and Development (OCED) countries said they trust their government in 2019.^2^ Distrust in politicians has been inconsistently associated with vaccine hesitancy, largely in Western countries [18, 19]. A recent poll found that 78% of respondents in the United States fear that the process for a COVID-19 vaccine approval has been more influenced by politics than science [20]. A European study of eight countries early in the pandemic (April 2–15, 2020) found that 55% of respondents were concerned about potential side effects of a vaccine [21]. An Ipsos poll on behalf of the World Economic Forum (July 24–August 7) surveyed countries regarding concerns about COVID-19 vaccine safety. Of the ten countries common to our study, 68% of the Swedish Ipsos respondents were concerned with safety. Our results suggest that trust in experts and confidence in health institutions can improve an individual’s trust in vaccines and reduce their risk perception about getting vaccinated. Steps are needed therefore to elevate those levels of trust and confidence. One way to accomplish this would be to build alliances across these experts and institutions to develop a non-political process for vaccine development that ensures safety and “high ethical standards” and includes the leaders of major drug development pharmaceutical companies [22].

Our cross-country analyses also illuminate the specific types of trust that seem to influence vaccine hesitancy by country, pointing toward possible country-specific solutions and emphasizing the need for additional, more comprehensive, country specific data. Our data illuminate the extent to which different types of leaders influence vaccine hesitancy in different countries. For example, in countries with a significant proportion of individuals who identify as Buddhists, including Thailand, Taiwan and Singapore, lack of trust in religious leaders was associated with responding “maybe” to taking a COVID-19 vaccine, but not with answering “no”. This is compared to Muslim-majority countries in our sample, where higher trust in religious leaders was associated with responding “maybe” in Indonesia and Malaysia but not in Turkey. Collecting country-specific data is especially important when attempting to design vaccination rollout strategies in low- and middle-income countries as there currently exists limited statistics on vaccine hesitancy within these countries.

In sum, our findings strongly suggest that trust in scientists and domestic healthcare professionals combined with confidence in the WHO are important drivers of vaccine acceptance across the globe. While there was some variation in levels of trust and attitudes toward the SARS-CoV-2 vaccine at the country level, lack of trust in health professionals and low confidence in domestic and international health institutions was consistently associated with vaccine hesitancy. As others have recognized [16], there is no singular approach to reducing vaccine hesitancy that has proven effective across time and place. However, these findings indicate that the best place to start for developing such an approach is boosting trust and confidence in these actors and institutions. When possible, political leaders should delegate management and communication of vaccine safety, effectiveness, and distribution protocols to scientists and health professionals. A study of vaccine hesitancy in Poland, for example, identified the strong role of health professionals in communicating about vaccines and disproving myths [23]. Health professionals should, in turn, enlist religious leaders when developing and deploying their communication strategies. Both political and religious leaders can be convinced to work closely with scientists and health professionals because they have incentives to minimize the magnitude of the pandemic’s social, political, and economic impact on their citizens and congregations, respectively. Moreover, both groups of leaders would reap long-term benefits. Public trust in science and confidence in the health ministry would reduce vaccine hesitancy and thereby help to bring the public health crisis more swiftly under control. The successful management of the pandemic would, in turn, bolster public trust in political leaders and religious leaders who were partners in these efforts.

## Data Availability

The raw data supporting the conclusion of this article will be made available by the authors, without undue reservation.

## Footnotes

1 Contact the authors for granular data on the distribution of trust and confidence measures across all countries.

2
http://www.oecd.org/gov/trust-in-government.htm
